# Erratum for Monsees et al., “Label-Free Raman Microspectroscopy for Identifying Prokaryotic Virocells”

**DOI:** 10.1128/msystems.00162-22

**Published:** 2022-03-10

**Authors:** Indra Monsees, Victoria Turzynski, Sarah P. Esser, André Soares, Lara I. Timmermann, Katrin Weidenbach, Jarno Banas, Michael Kloster, Bánk Beszteri, Ruth A. Schmitz, Alexander J. Probst

**Affiliations:** a Group for Aquatic Microbial Ecology, Environmental Microbiology and Biotechnology, University Duisburg-Essen, Essen, Germany; b Institute for General Microbiology, Christian Albrechts University, Kiel, Germany; c Phycology Group, Faculty of Biology, University Duisburg-Essen, Essen, Germany; d Centre of Water and Environmental Research (ZWU), University of Duisburg-Essen, Germany; e Essen, Germany

## ERRATUM

Volume 7, no. 1, e01505-21, 2022, https://doi.org/10.1128/mSystems.01505-21. On page 11, since original publication of this article, a name has been removed from Acknowledgments at the person’s request.

