# Supplementary figures and images for: Label-Free Raman Microspectroscopy for Identifying Prokaryotic Virocells
Source: mSystems. 2022 Feb 15;7(1):e01505-21. doi: 10.1128/msystems.01505-21 (PMC8845568; doi:10.1128/msystems.01505-21)

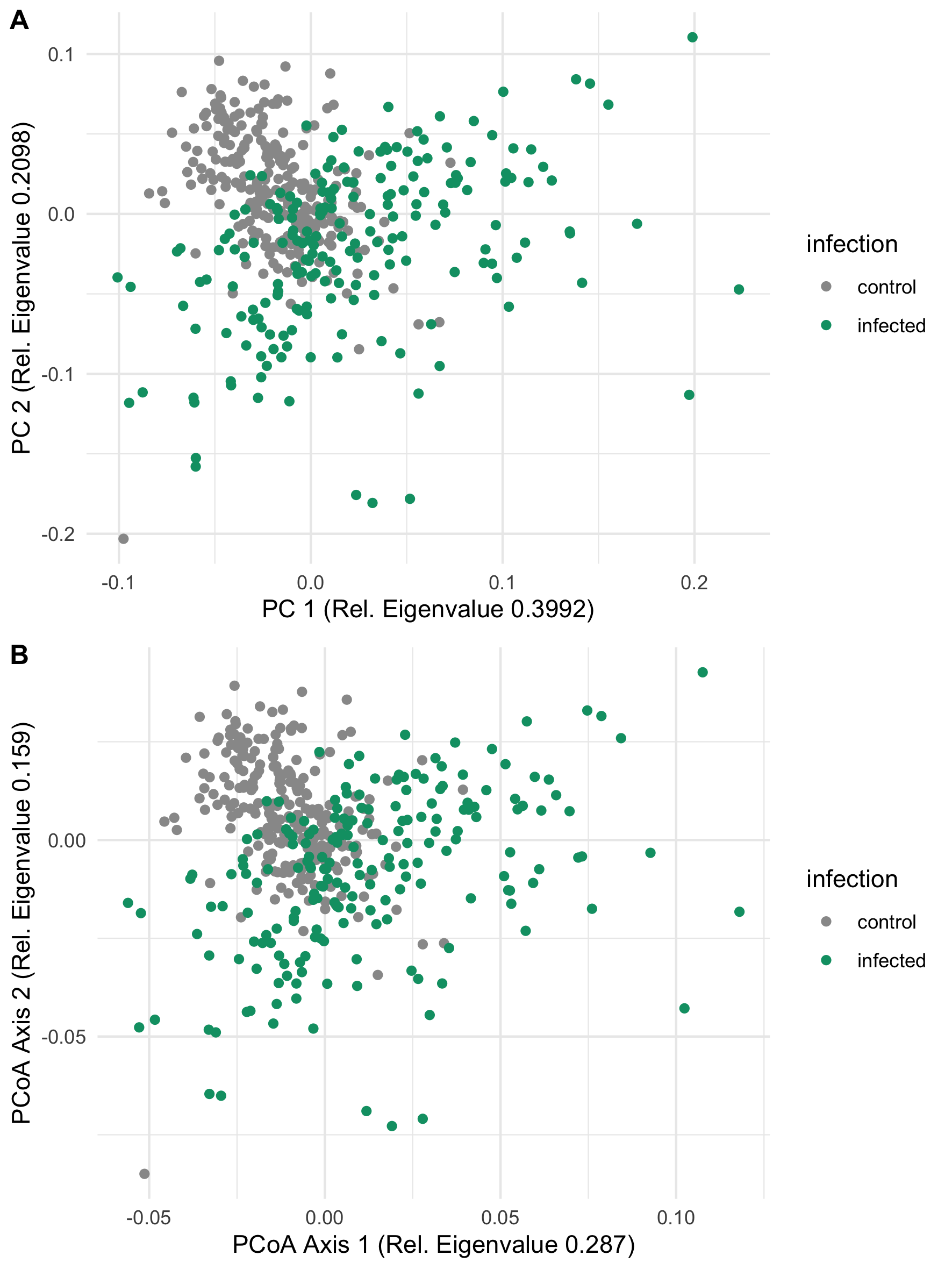

Supplement: FIG S1 [file msystems.01505-21-sf001.tif]

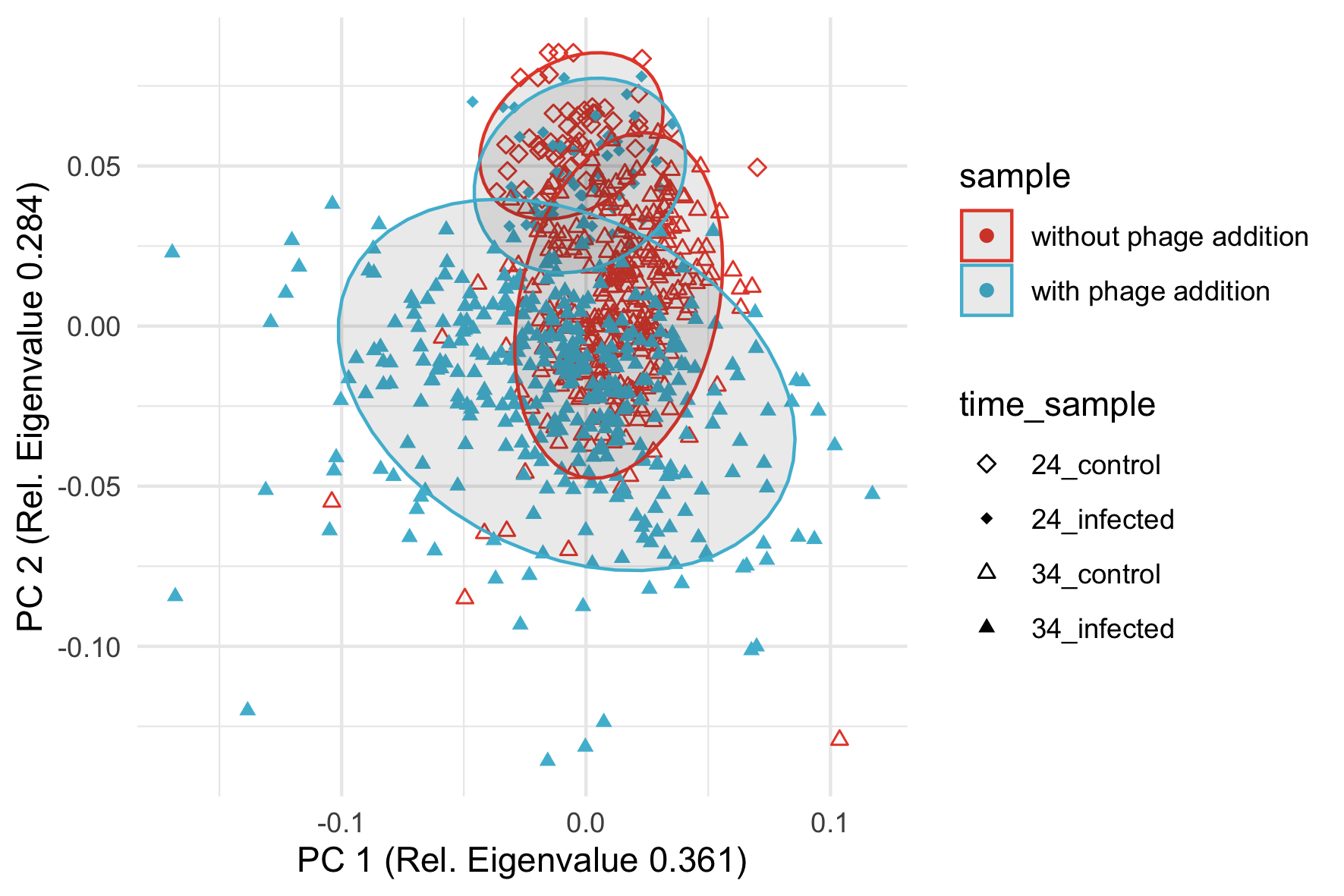

Supplement: FIG S2 [file msystems.01505-21-sf002.tif]

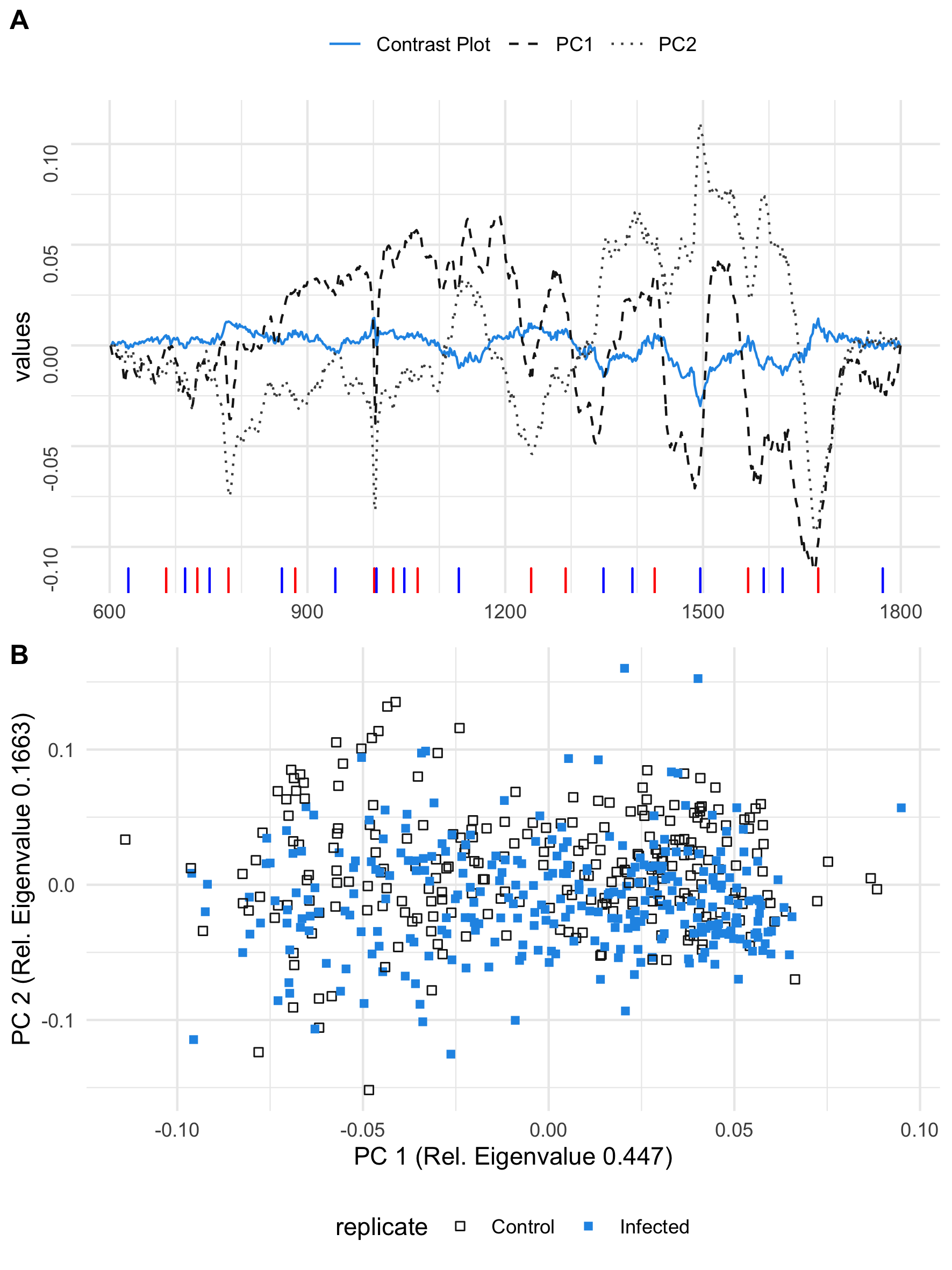

Supplement: FIG S3 [file msystems.01505-21-sf003.tif]

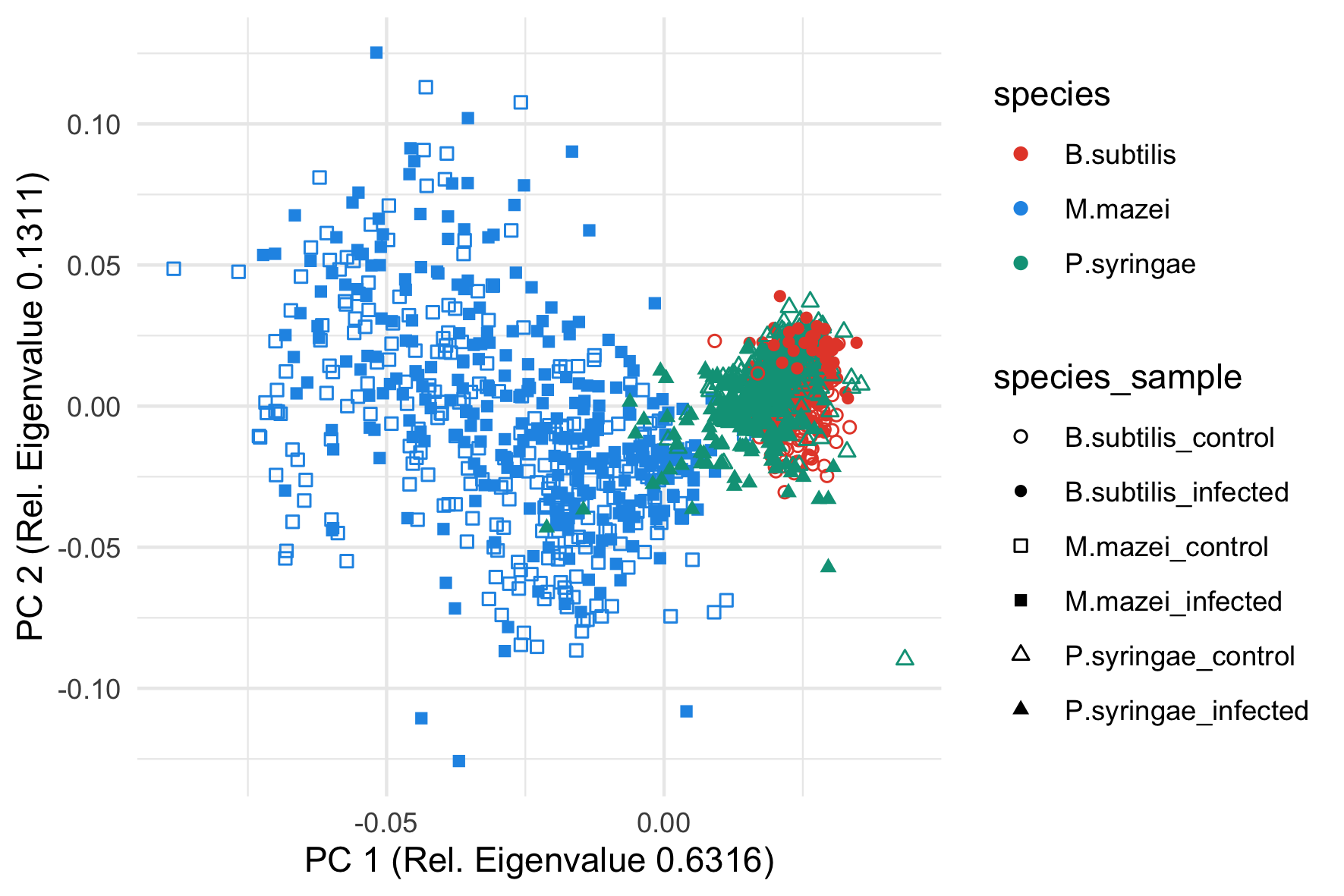

Supplement: FIG S4 [file msystems.01505-21-sf004.tif]

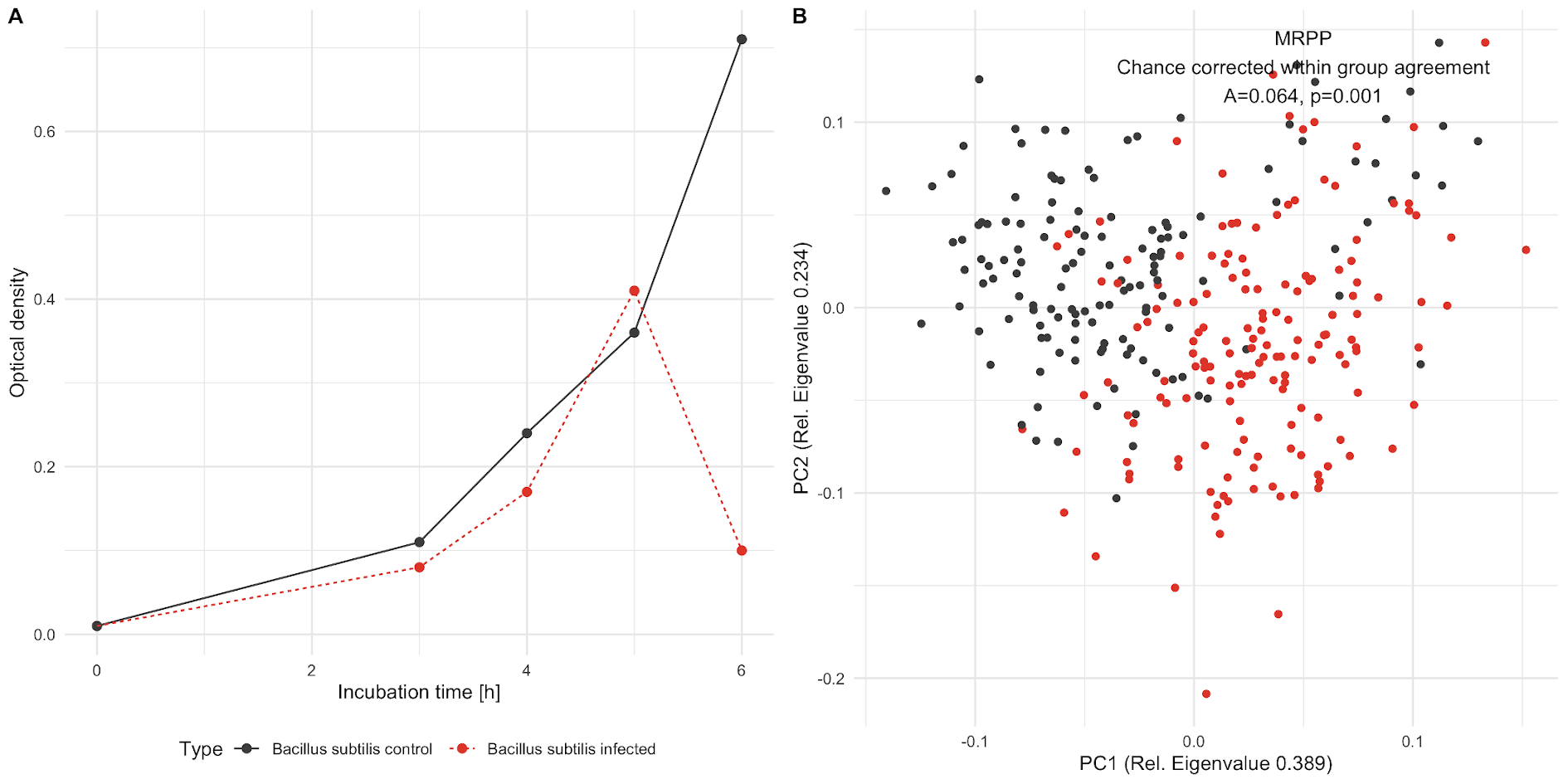

Supplement: FIG S5 [file msystems.01505-21-sf005.tif]

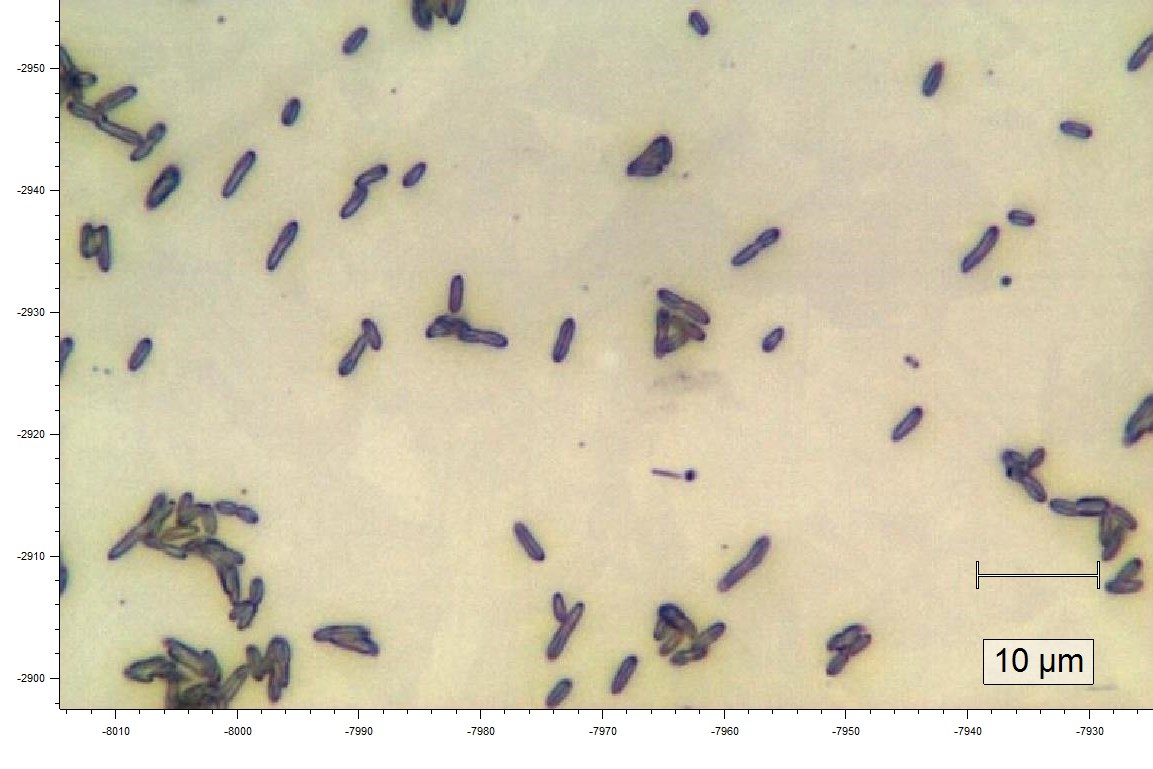

Supplement: FIG S6 [file msystems.01505-21-sf006.jpg]
