# Supplementary material for: Label-Free Raman Microspectroscopy for Identifying Prokaryotic Virocells
Source: mSystems. 2022 Feb 15;7(1):e01505-21. doi: 10.1128/msystems.01505-21 (PMC8845568; doi:10.1128/msystems.01505-21)
Supplement: TABLE S1 [file msystems.01505-21-st001.pdf]

Species	virus addition	number of spectra
Pseudomonas syringae	no	250
Pseudomonas syringae	yes	198
Bacillus subtilis	no	135
Bacillus subtilis	yes	159
Methanosarcina mazei	no	251
Methanosarcina mazei	yes	294
