# Supplementary material for: Label-Free Raman Microspectroscopy for Identifying Prokaryotic Virocells
Source: mSystems. 2022 Feb 15;7(1):e01505-21. doi: 10.1128/msystems.01505-21 (PMC8845568; doi:10.1128/msystems.01505-21)
Supplement: TABLE S2 [file msystems.01505-21-st002.pdf]

Samples	Dunn (a=0.05)
***Bacillus subtilis* infected-<br>*Bacillus subtilis* control**	4.851780<br>0.0000*
Methanosarcina mazei control-<br>*Bacillus subtilis* control	4.557990<br>0.0000*
Methanosarcina mazei control-<br>*Bacillus subtilis* infected	10.36254<br>0.0000*
Methanosarcina mazei infected-<br>*Bacillus subtilis* control	3.311813<br>0.0005*
Methanosarcina mazei infected-<br>*Bacillus subtilis* infected	9.234893<br>0.0000*
***Methanosarcina mazei* infected-<br>*Methanosarcina mazei* control**	1.653004<br>0.0492
Pseudomonas syringae control-<br>*Bacillus subtilis* control	0.903970<br>0.1830
Pseudomonas syringae control-<br>*Bacillus subtilis* infected	6.531711<br>0.0000*
Pseudomonas syringae control-<br>*Methanosarcina mazei* control	4.352914<br>0.0000*
Pseudomonas syringae control-<br>*Methanosarcina mazei* infected	2.872524<br>0.0020*
Pseudomonas syringae infected-<br>*Bacillus subtilis* control	9.301452<br>0.0000*
Pseudomonas syringae infected-<br>*Bacillus subtilis* infected	15.03088<br>0.0000*
Pseudomonas syringae infected-<br>*Methanosarcina mazei* control	-5.783991<br>0.0000*
Pseudomonas syringae infected-<br>*Methanosarcina mazei* infected	-7.532194<br>0.0000*
***Pseudomonas syringae* infected-<br>*Pseudomonas syringae* control**	-9.876491<br>0.0000*
