# Supplementary material for: Label-Free Raman Microspectroscopy for Identifying Prokaryotic Virocells
Source: mSystems. 2022 Feb 15;7(1):e01505-21. doi: 10.1128/msystems.01505-21 (PMC8845568; doi:10.1128/msystems.01505-21)
Supplement: TABLE S3 [file msystems.01505-21-st003.pdf]

Cell.ID	Ratio
ps_34_N13_St_30_9_01	excluded
ps_34_N13_St_30_9_028	excluded
ps_34_N13_St_30_9_032	excluded
ps_34_N13_St_30_9_26	1.0013
ps_24_N14_St_25_8_5	excluded
ps_24_N14_St_25_8_13	0.9698
ps_24_N14_St_25_8_18	0.8600
ps_24_N14_St_25_8_27	0.9132
ps_24_N14_St_25_8_28	0.7925
ps_24_N14_St_25_8_35	0.8021
ps_24_N14_St_25_8_8	0.76
